# Scale-up production of and dietary supplementation with the recombinant antimicrobial peptide tilapia piscidin 4 to improve growth performance in *Gallus gallus domesticus*

**DOI:** 10.1371/journal.pone.0253661

**Published:** 2021-06-24

**Authors:** Hsueh-Ming Tai, Ming-Feng You, Chia-Hua Lin, Tsung-Yu Tsai, Chieh-Yu Pan, Jyh-Yih Chen

**Affiliations:** 1 Marine Research Station, Institute of Cellular and Organismic Biology, Academia Sinica, Jiaushi, Ilan, Taiwan; 2 Department and Graduate Institute of Aquaculture, National Kaohsiung University of Science and Technology, Kaohsiung, Taiwan; National Cheng Kung University, TAIWAN

## Abstract

Antimicrobial peptides (AMPs) are short and positively charged peptides with broad-spectrum antimicrobial activities. AMPs have been investigated as potential antibiotic alternatives to improve growth performance and prevent pathogen infection in the poultry industry. The antimicrobial peptide tilapia piscidin 4 (TP4) was derived from *Oreochromis niloticus*, possesses antimicrobial activities and immunomodulatory properties, promotes intestinal health, and protects against pathogen infection. The codon-optimized sequence of TP4 was introduced into the pPICZαA vector and transformed into *Pichia pastoris*. Large-scale expression was induced following culture with methanol in a 500-liter fermenter. Freeze drying of fermented rTP4 broth and then rTP4 evaluation as a feed additive for *Gallus gallus domesticus* were performed. The *in vitro* antimicrobial activity of recombinant TP4 (rTP4) against gram-positive and gram-negative pathogens was evaluated. Evaluation of the effect of temperature on the antimicrobial activity of rTP4 showed its high stability at high temperatures. rTP4 significantly enhanced the phagocytic activity of macrophage cells, indicating that rTP4 has a remarkable ability to stimulate macrophages. rTP4 was used as a dietary supplement at 0.75, 1.5, 3.0, 6.0 and 12% in *G*. *g*. *domesticus* for five weeks, and growth performance, gut microbiota composition, and histology were assessed. The 3.0% rTP4 supplement group showed a significant increase in weight gain ratio and feed efficiency compared to those of the basal broiler diet group. Crude rTP4 was expressed by yeast to significantly promote growth efficiency and resistance against pathogens in *G*. *g*. *domesticus*, which could indicate its use as a suitable alternative to antibiotics as feed additives in the poultry industry.

## 1. Introduction

Antibiotics are commonly used to prevent disease and promote growth in agriculture. For poultry and livestock production, antibiotics are often used to treat clinical diseases and to control and prevent common infections. While antibiotics are used in animals for growth promotion or increased feed efficiency [1], the low concentration of antibiotics to which bacteria are exposed for a long period of time is problematic. A random mutation could cause some bacteria to become resistant to antibiotics. Drug-resistant microbes can survive during the antibiotic treatment period, and their population can be increased through natural selection. In recent decades, antibiotics have been used in poultry production as therapeutic agents to treat and control necrotic enteritis and respiratory diseases and to improve feed conversion and body weight gain [2]. These effects are due mainly to antibiotic treatment-induced gut microbiota alteration and microbiota modification in the gastrointestinal tract to provide an optimal microbiota for growth [3]. However, unlimited use of antibiotics will lead to reduced therapeutic effectiveness and generate many drug-resistant strains that may then have negative effects on animal and human health [4].

According to published data, different antibiotic‐resistant microbial strains have emerged, and the incidence of diseases caused by drug-resistant bacterial infection is increasing worldwide. There is an urgent need to study and introduce new types of effective, safe and natural compounds. Among these antimicrobial materials, AMPs have great potential as excellent alternatives to routine antibiotics used in the porcine and fowl industries [5]. Antimicrobial peptides possess extensive antimicrobial activities to inhibit gram-negative and gram-positive pathogens, fungi, viruses, and cancerous cells. Natural or artificial AMPs have been indicated to prevent bacterial colonization of cell surfaces, kill or inhibit microbes in biofilms, and destroy biofilm structures [6].

Most antimicrobial peptides are cationic peptides with conserved amphipathic molecular structure motifs that confer antibacterial properties [7]. Bacterial defense peptides were initially isolated from mammalian neutrophil granules, insect lymph and the skin of splendid leaf frogs [8] and demonstrated to have antimicrobial activities. Cationic defense peptides usually have few amino acids and are hydrophobic and hydrophilic charged [9]. The AMPs are then able to interact with the cytoplasmic membrane and rapidly destroy essential components inside the cells. Piscidins are histidine-enriched and linear antimicrobial peptides in the AMP family. The histidine content of piscidins and amphipathicity of peptides can cause different orientations of membrane insertion and permeability that very with pH value [10]. Piscidins isolated from teleost fish taxa consisting of 18 to 46 amino acids are expanded teleost-specific AMPs [11], which have a wide range of biological functions, including antibacterial, antiviral, antiparasite, antitumor, and immunomodulatory functions [12–16].

The antimicrobial peptide piscidin was overexpressed in *Escherichia coli*, recovered from inclusion bodies and purified by Ni-NTA chromatography [17–19]. However, in a prokaryotic expression system, expressed heterologous eukaryotic proteins may not be efficiently secreted into the broth and may fold incorrectly in large quantities. Yeast have been used to produce eukaryotic proteins in large amounts and active forms that are secreted into the medium. The antimicrobial peptide tilapia piscidin 4 (TP4) was derived and identified from *Oreochromis niloticus* and has activity against a broad range of microbial strains [20–22]. In this study, a crude extract of a recombinant tilapia piscidin 4 (rTP4) culture was expressed by *P*. *pastoris* to obtain antimicrobial properties, which were tested against gram-positive and gram-negative pathogens of chickens and ducks. Simultaneously, the effect of dietary supplementation with the antimicrobial peptide rTP4 on the growth efficiency of *G*. *g*. *domesticus* was evaluated.

## 2. Methods

### 2.1 Microorganisms and animals

The bacterial strain *Pichia pastoris* X-33 (Invitrogen, CA, USA) was used as the TP4 expression host. *Escherichia coli* BL21 (DE3) (Stratagene, La Jolla, Calif., USA) was used as a DNA donor. DNA encoding TP4 with a His-tag was ligated into the pPICZαA (cat. no. V195-20, Invitrogen, CA, USA) expression vector. Procedures involving these animals adhered to the requirements of National Pingtung University of Science and Technology (NPUST) regarding their health and welfare and were approved by the Institutional Animal Care and Use Committee of NPUST (NPUST-107-026).

### 2.2. Construction and transformation of the expression recombinant plasmid and selection of positive transformants

To further increase the protein expression of recombinant TP4 in *P*. *pastoris*, the codon-optimized TP4 gene was designed to match *P*. *pastoris* expression by artificial gene synthesis from Omics Bio (Taipei, Taiwan). The nucleotide sequence products were confirmed by sequencing using primers, and then the identified plasmid was digested with EcoRI and XbaI. After enzyme digestion, the insert was ligated into the pPICZαA vector. The pPICZαA plasmid ligated with TP4 was digested with PmeI to linearize the vector and then transformed into *P*. *pastoris* X-33 by electroporation. The *P*. *pastoris* X-33 strain integrated with recombinant pPICZαA-TP4 DNA was grown in YPD broth at 30°C with 250 rpm agitation for 16 h. The resulting culture was inoculated into 30 mL of BMGY medium and incubated for 24 h at 30°C. The *P*. *pastoris* culture was then harvested by 10 min of centrifugation at 3,000 xg. The cell pellet was resuspended in 20 mM phosphate buffer, sonicated (400 cycles with 3 sec on/3 sec off) on ice by using a Sonicator 3000 system (HEAT Systems Inc., Farmingdale, NY) and then centrifuged at 5,000 xg for 20 min at 4°C. The recombinant protein in the pellet and supernatant was detected by SDS-PAGE and Western blotting using an anti-6x-His tag monoclonal antibody (Invitrogen, MA1-21315).

### 2.3. Scale-up production of recombinant protein in a 500-L fermenter

The stock was streaked on YPD agar plates with zeocin and incubated for two days at 30°C. BMGY with PTM4 broth and zeocin (100 μg/mL) was inoculated with a single colony and incubated in a shaker at 30°C and 150 rpm for two days. A second preculture consisting of 5% cultured broth was inoculated into YPD medium under the same conditions for one day. In this experiment, a 500-liter fermenter (BTF-C500L, Biotop Process & Equipment Inc, Nantou, Taiwan) was used with 350 liters of commercial medium. Fermentor cultivations were carried out by using basal salt medium and PTM1. During the fermentation period, the temperature was maintained at 30°C, and the pH was maintained and monitored at 6.0 with the addition of 14.5% ammonia and 0.1 N H_2_SO_4_. During the cultivation phase, the yeast were allowed to grow until the glycerol was exhausted after 36.5 h. Subsequently, the glycerol fed-batch phase consisted of feeding 50% (w/v) glycerol (containing 12 mL/L PTM) into the fermenter. After 47 h of cultivation, the methanol induction phase was performed by feeding 100% methanol (containing 16 mL/L PTM) for 24 h to induce rTP4 production. The rTP4-expressing yeast broth was harvested and freeze dried. Before rTP4 mixing with fodder preparation, rTP4 expression in the fermented culture and spray-dried powder was confirmed by Western blot analysis, and the rTP4 concentration was measured by comparing with different concentrations of synthetic TP4 peptide.

### 2.4. Antimicrobial activity of rTP4

The antimicrobial activity of rTP4 was measured in cultures of *Escherichia coli* (BCRC 10675), *Pseudomonas aeruginosa* (ATCC 19660), *Staphylococcus aureus* (BCRC 10780), and *Riemerella anatipestifer* (RA9, CFC27, CFC437, RA3, CFC363 and RA16). The microorganisms were collected from a single expanded colony and stored at -80°C. The microorganisms were inoculated in liquid broth and incubated at 37°C on a rotary shaker at 150 rpm overnight. The microorganisms were then diluted into fresh medium (1:1000) and incubated at 37°C on a rotary shaker. The bacterial cultures (10^4^ CFU/ml) were mixed with 100 μl of an rTP4 solution and incubated at 37°C overnight. A negative control consisted of supernatant from *P*. *pastoris* transformed with vector alone, and ampicillin (2 mg/mL) was used as a positive control. After a 24-h incubation, the culture broth was measured at a wavelength of 600 nm. The antimicrobial activity of rTP4 was tested with an inhibition zone assay at different treatment temperatures [23]. Experiments were performed in duplicate and repeated independently three times.

### 2.5. *G*. *g*. *domesticus* maintenance and dietary treatment

A total of 119 male and 119 female 2-day-old *G*. *g*. *domesticus* were equally divided into seven groups for different dietary treatments, including basal diet, Spiraline-A (Juily Pharmaceutical Co., Ltd. San Sia, Taipei Hsien, Taiwan) and rTP4 groups (0.75, 1.5, 3.0, 6.0 and 12%). Thirty-four *G*. *g*. *domesticus* were housed in each cage with different treatment groups. The formulation of the basal diet and the composition analysis of the basal feed with additive in the early, middle and late stages are presented in S1 and S2 Tables. The chickens had *ad libitum* access to water and fodder for the duration of the trial from 3 to 36 days of age. The animals were kept in a 12:12 light-dark cycle at 18.4–31.2°C in a cage with a constant environment. The rTP4 feed, fecal sludge and wastewater were not discarded directly into the environment. Statistical analysis was performed with Student’s t-test when comparing two groups. Multiple group comparisons were performed by ANOVA in GraphPad Prism 9. *P ≤ 0.05, **P ≤ 0.01, ***P ≤ 0.001, ****P ≤ 0.0001.

### 2.6. The analysis of the phagocytic activity and cell size and complexity assessments

The effect of rTP4 on phagocytic activity was studied with the use of GFP-tagged *E*. *coli* bacteria using flow cytometry. The phagocytosis of GFP-*E*. *coli* (ATCC 25922GFP™) was analyzed by monitoring the mean GFP fluorescence intensity of engulfed bacteria. RAW264.7 cells were preincubated with rTP4 in 10% FBS/DMEM with gentle shaking for 24 h at 37°C. The rTP4-induced RAW264.7 macrophage cells were allowed to uptake and become infected with GFP-labeled *E*. *coli* (10^7^ cfu/mL) at an infective concentration of 1:20 (MOI) for an additional 4 h. The change in phagocytic activity was assessed according to the change in fluorescence intensity. After rTP4 stimulation, cells were removed from the culture dishes using a cell scraper and then centrifuged and washed with PBS. The cells were resuspended in a PBS solution. Flow cytometer (Beckman Coulter) analysis was performed using forward (FSC: cell size) and side scatter (SSC: cell complexity) parameters.

### 2.7. Physiological analysis and histological procedures

During the experimental period, the feed intake, body weight and survival rate were measured. Weight gain (WG), protein efficiency ratio (PER), feed efficiency (FE), and survival percentage were calculated. After 36 days of the feeding trial, *G*. *g*. *domesticus* were euthanized, and blood serum samples were collected after centrifugation (3,000 ×*g* for 10 min) and stored at -80°C for analysis. The specimens were fixed in 4% formaldehyde in 0.1 M sodium phosphate buffer (PBS, pH 7.2) at room temperature. Subsequently, they were rinsed in PBS and dehydrated in graded ethanol. Tissue sections were deparaffinized in xylene and hydrated using serial percentages of alcohol. The sections were stained with hematoxylin for 10 min and eosin for 5 min.

### 2.8. Gut microbiota analysis

The duodenum stool of *G*. *g*. *domesticus* fed with the basal, Spiraline-A, or rTP4 diet was dissected for genomic DNA extraction using an innuPREP stool DNA kit (Jena AG, Jena, Germany). The DNA concentration was measured by using a NanoDrop ND1000 spectrophotometer (NanoDrop® Technologies), and DNA was stored at -20°C until use. The V4 region of the 16S rRNA gene was amplified with the forward PCR primer 515F and reverse primer 806R. All samples were amplified on a Veriti 96-well Thermal Cycler (Applied Biosystems, Foster City, USA). PCR amplicon sequencing was performed by the Biotools Microbiome Research Center (New Taipei City, Taiwan). The sequences were then processed using QIIME software [24]. QIIME can perform taxonomic assignment at the genus level on the sequencing from the Biotools Microbiome Research Center.

## 3. Results

### 3.1. Construction of a TP4 expression pPICZαA plasmid

**Fig 1:**
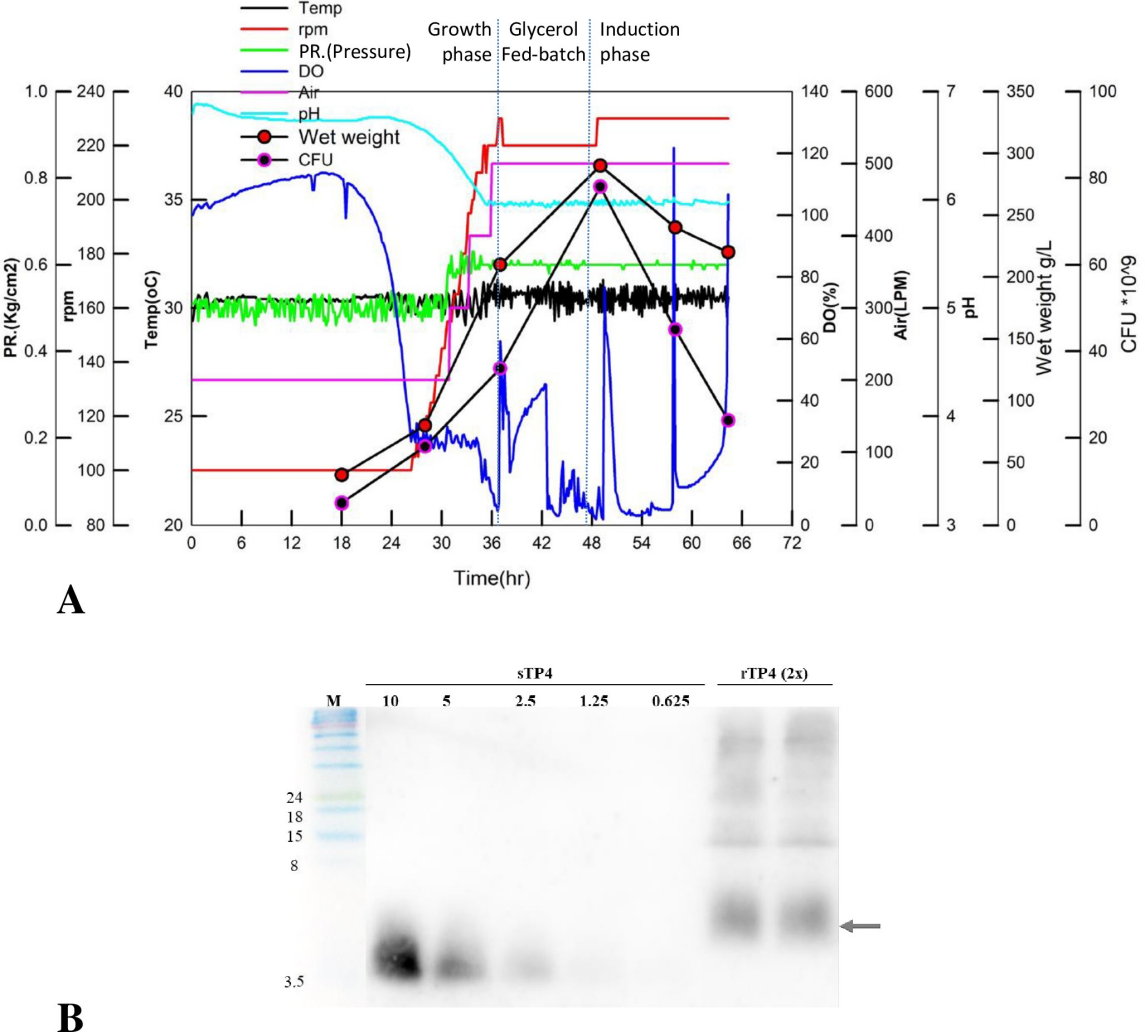
Scale-up expression of rTP4 in *P*. *pastoris* in a 500-liter fermenter. (A) The dissolved oxygen (%), pH, RPM and temperature (°C) of broth during incubation of a *P*. *pastoris* X-33 transformant in BSM. (B) Western blot analysis profiles of recombinant rTP4 expression. M: Molecular weight marker; sTP4: Synthetic TP4 with concentrations of 10, 5, 2.5, 1.25 and 0.635 ng/μL; rTP4: Recombinant TP4.

DNA encoding TP4 with a 6xHis tag was inserted into the pPICZαA expression vector to obtain the α-Factor-TP4-6xHis expression cassette (Fig 1). The pPICZαA plasmid ligated with TP4 was digested with PmeI within the AOX1 region to linearize the pPICZαA vector, and then TP4 genes were transformed into the *Pichia pastoris* X-33 strain. Colonies were screened by plating on YPDS plates containing different concentrations of zeocin. Transformed *P*. *pastoris* X-33 colonies integrated with recombinant pPICZαA-TP4 DNA were then selected by colony hybridization by using an anti-6xHis tag antibody, and high-expression clones were picked for subsequent experiments (previous data). The transformants were confirmed by colony PCR analysis with AOX1 primers (previous data).

### 3.2. Construction of the TP4 expression pPICZαA plasmid and large-scale expression of recombinant TP4 in a 500-L fermenter

**Fig 2:**
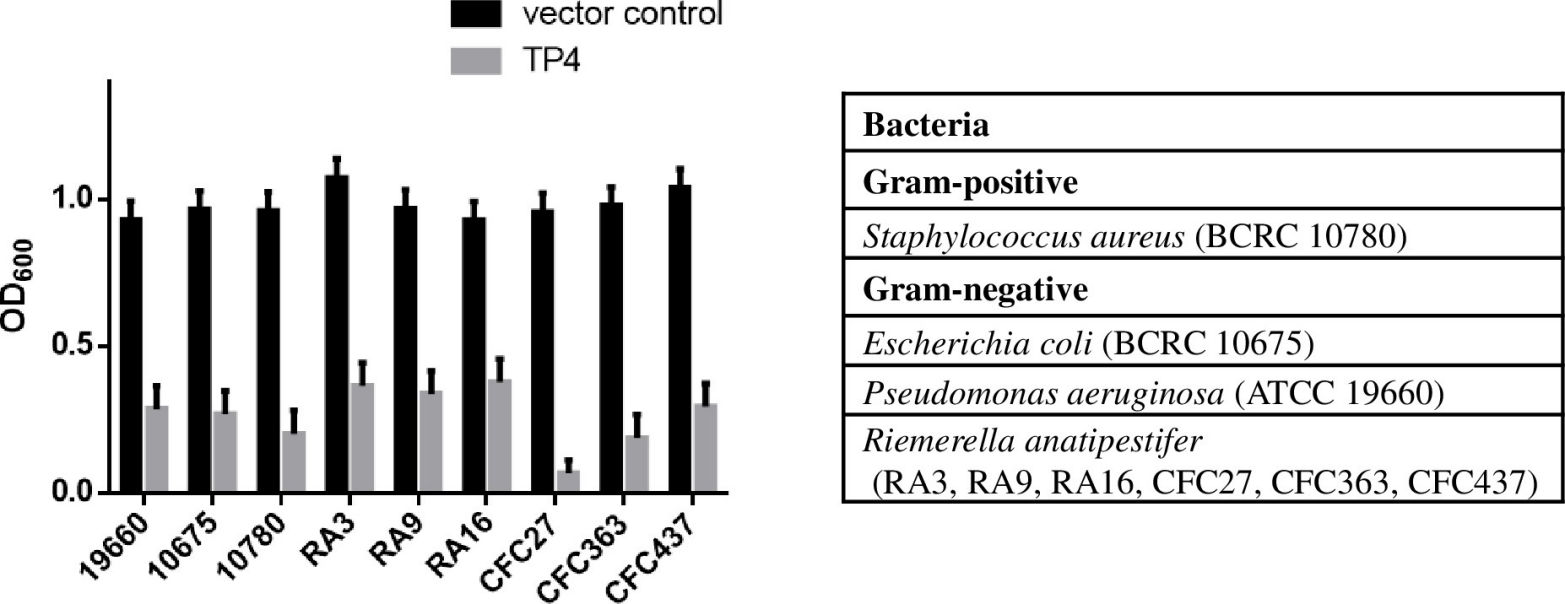
The effects of rTP4 on gram-negative and gram-positive strains by coincubation assay. After incubation, the culture medium was measured at a wavelength of 600 nm. A low OD_600_ value indicates that bacterial growth was inhibited. Vector control: Empty pPICZαA vector. TP4: Protein expressed by the pPICZαA-TP4 vector.

DNA encoding TP4 with a 6xHis tag was inserted into the pPICZαA expression vector to obtain the α-Factor-TP4-6xHis expression cassette, which was described in our previous report [25]. Recombinant TP4 was expressed in the methylotrophic yeast *P*. *pastoris* under the control of the *AOX1* promoter and secreted into the broth using the α-factor pre-pro sequence with the *STE13* gene for dipeptidyl aminopeptidase A (Fig 1). After the successful production of the rTP4 transformant, the cells were cultured in a 5-liter bench-top fermenter. The parameters of dissolved oxygen (DO), RPM, pH and agitation were optimized for rTP4 production, and the effect of induction time of rTP4 expression was evaluated (previous data). We enlarged the scale from a 5 to 500 L fermenter with a two-stage fed-batch cultivation. Five percent of a *P*. *pastoris* culture was inoculated into 350 L of BSM. The cultivation process is shown in Fig 2A. During 36.5 h of cultivation, glycerol content decreased with time, and we began to feed glycerol into the fermentation. When the *P*. *pastoris* transformant was growing to stationary phase, the highest viable count reached approximately 8.3×10^10^ CFU/mL at 48 h of cultivation. At the same time, methanol inductions were carried out in which the viable count and wet weight of yeast showed a sharp decrease (Fig 2A). After 65 h of cultivation, the fermented broth was harvested and then freeze dried to powder. The rTP4 powder was confirmed by Western blot analysis, and the rTP4 concentration was measured at 1.07 mg/g by comparison with different concentrations of synthetic TP4 peptide (Fig 2B). rTP4 powder was used as a feed additive for dietary supplementation in *G*. *g*. *domesticus*.

### 3.3. Antimicrobial activity of rTP4 expression in *P*. *pastoris*

**Fig 3:**
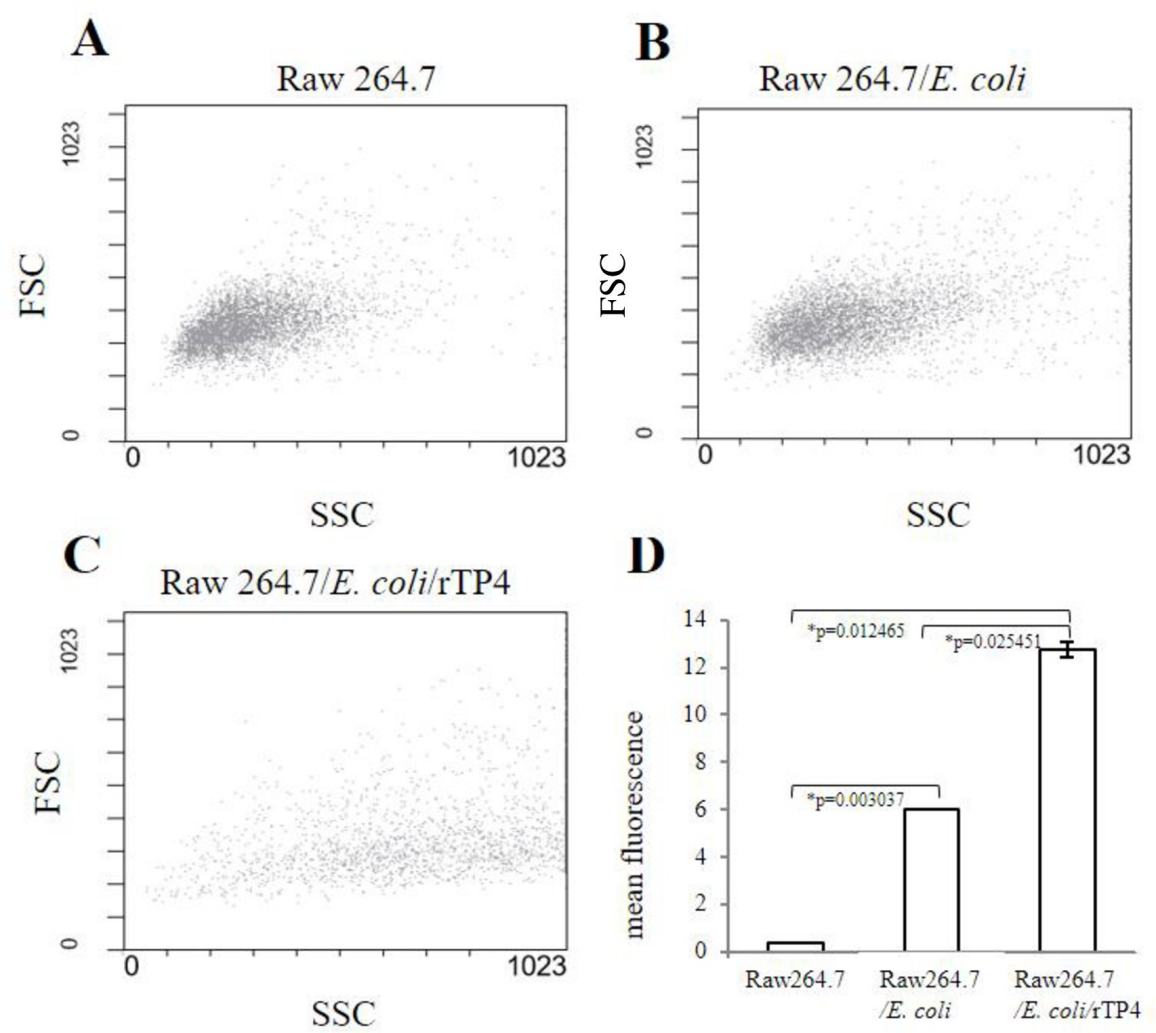
Flow cytometry phagocytosis assay. Flow cytometry analysis using cell size forward-scattered light (FSC) and side-scattered light (SSC) parameters was performed. (**A**) RAW264.7 cells alone, (**B**) RAW264.7 cells coincubated with *E*. *coli*, and (**C**) RAW264.7 cells treated with rTP4 and coincubated with *E*. *coli*. (**D**) The mean fluorescence intensity (MFI) of RAW264.7 cells was analyzed, and the asterisks indicate significant differences.

To evaluate the antimicrobial activity of the rTP4 protein, the culture was induced with methanol and mixed with equal volumes of diluted bacterial cultures of gram-positive and gram-negative pathogens. After 16 h of incubation, the culture medium was measured at a wavelength of 600 nm. Compared to *P*. *pastoris* with a vector control, which did not inhibit pathogen growth, rTP4 showed high activity to inhibit pathogen growth (Fig 3). Meanwhile, the Kirby-Bauer disk diffusion method was used for antimicrobial susceptibility testing of rTP4, and the results indicated that rTP4 had a larger diameter and a more visible zone against the tested strains than the control treatment. The antimicrobial peptide was most effective against the pathogens *R*. *anatipestifer* (RA3) and *R*. *anatipestifer* (CFC27) (Table 1A). Furthermore, a study of the thermal stability of rTP4 was performed against *S*. *aureus* (BCRC 10780), *P*. *aeruginosa* (ATCC 19660) and *E*. *coli* (BCRC 10675). The thermal stability of the antimicrobial peptide was tested at 40, 60, 80 or 100°C for 5 min, and the selected pathogens were detected by the agar disk diffusion method. The negative control group was *P*. *pastoris* with the empty vector. The antimicrobial peptide rTP4 showed good temperature stability at high temperature, inhibiting *S*. *aureus* and *E*. *coli* growth. The *P*. *aeruginosa* strain was inhibited by the antibacterial activity of rTP4 with increasing temperature (Table 1B). The results showed that heat treatment of rTP4 did not significantly affect the antimicrobial ability at temperatures up to 100°C.

**Table 1:** The antimicrobial activity and effect of temperature of rTP4. The rTP4 was expressed by *P*. *pastoris* X-33 and supernatant were collected after induction and applied to disk diffusion assay. After incubation, the inhibition zone diameters were measured on the culture plate. Representative radial diffusion assays are shown. (A) The antimicrobial activity of rTP4. (B) The effect of temperature on rTP4 antimicrobial activity.

**A.** The inhibition zone diameter methodological analysis of the effect of fermentation supernatants			
Microorganism	Inhibition zone diameter (mm)^a^		
	Vector control	rTP4	Ampicillin (2 mg/ml)
*Escherichia coli *(BCRC 10675)	NI*	9.6±1.1	13.6±0.5
*Pseudomonas aeruginosa* (ATCC 19660)	NI	10.3±0.5	11.6±0.5
*Staphylococcus aureus* (BCRC 10780)	NI	10.6±1.1	14.6±0.5
*Riemerella anatipestifer* (RA9)	NI	NI	31±0.8
*Riemerella anatipestifer* (CFC27)	NI	15.2±0.5	43±1.1
*Riemerella anatipestifer* (CFC437)	NI	13.6±0.3	15±4.5
*Riemerella anatipestifer* (RA3)	NI	14.2±0.5	12±2.5
*Riemerella anatipestifer* (CFC363)	NI	13.2±0.2	32±2.3
*Riemerella anatipestifer* (RA16)	NI	NI	30±1.2

**B.** The effect of temperature on rTP4 antimicrobial activity				
Temperature	Inhibition zone diameter (mm)			
	Vector control^2^	*Staphylococcus aureus* (BCRC 10780)	*Pseudomonas aeruginosa* (ATCC 19660)	*Escherichia coli* (BCRC 10675)
40°C	NI^1^	11.0±1.0	11.6±1.5	9.6±1.5
60°C	NI	10.6±0.5	10.6±1.1	9.6±0.5
80°C	NI	10.3±0.5	9.1±0.7	9.5±0.8
100°C	NI	9.8±0.2	NI	9.5±0.5

^1^NI, no inhibition.

^2^ Vector control was fermentation supernatant from transformants harboring empty plasmid. Ampicillin (2 mg/ml) was applied at a volume of 10 μl to disk paper.

### 3.4. Effects of rTP4 on cell size and complexity in a phagocytic activity assay

To evaluate rTP4 *in vitro* stimulation of macrophage differentiation, the RAW264.7 cell line was treated with rTP4 for 24 h. Flow cytometry analysis using cell size forward-scattered light (FSC) and side-scattered light (SSC) parameters was performed. The results showed that the cell size and complexity after GFP-*E*. *coli* phagocytosis by RAW264.7 cells without rTP4 treatment were similar to those of RAW264.7 cells alone (Fig 3A and 3B). After rTP4 stimulation, the immunogenicity increased with molecular size and complexity in the macrophage differentiation assay (Fig 3C). In addition, increased cell complexity may be associated with increased storage or secretory vesicles, such as lysosomes. The mean fluorescent intensity (MFI) showing phagocytosis of GFP-*E*. *coli* in RAW264.7 cells after treatment with rTP4 was higher than that in the other groups (Fig 3D).

### 3.5. rTP4 supplementation improves growth performance and histology

**Fig 4:**
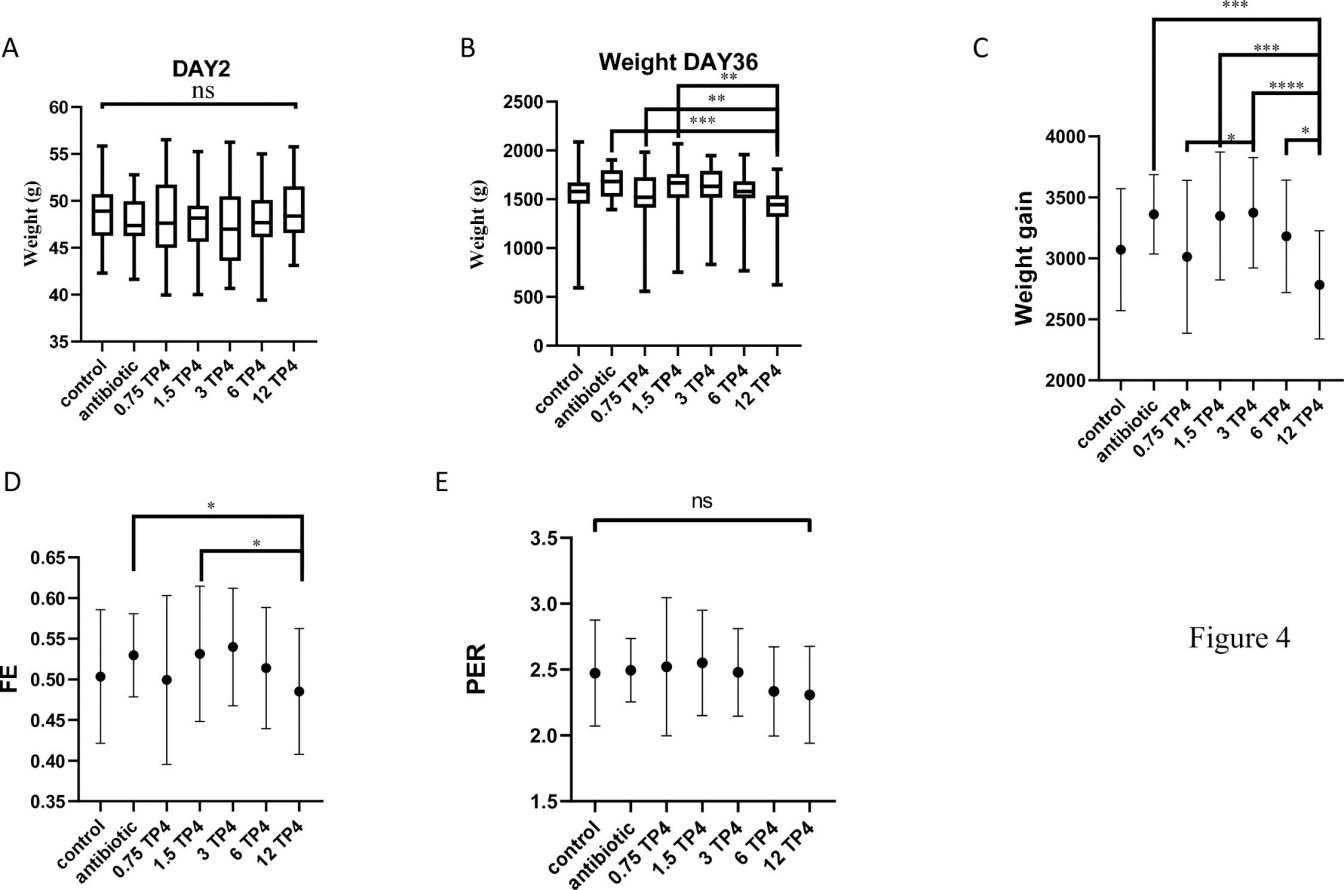
Effect on feed parameter analysis. (A) Weight for day 2 and (B) day 36 analysis between groups. (C) weight gain, (D) FE (feed efficiency), and (E) PER (protein efficiency ratio) of *G*. *g*. *domesticus* fed diets containing rTP4 (0.75, 1.5, 3.0, 6.0 or 12%) spray-dried powder for 5 weeks. Each bar represents the mean value from three determinations, with the standard error (SE). Data (mean ± SE) with star marker represent differ significantly (P<0.05) among treatments.

**Fig 5:**
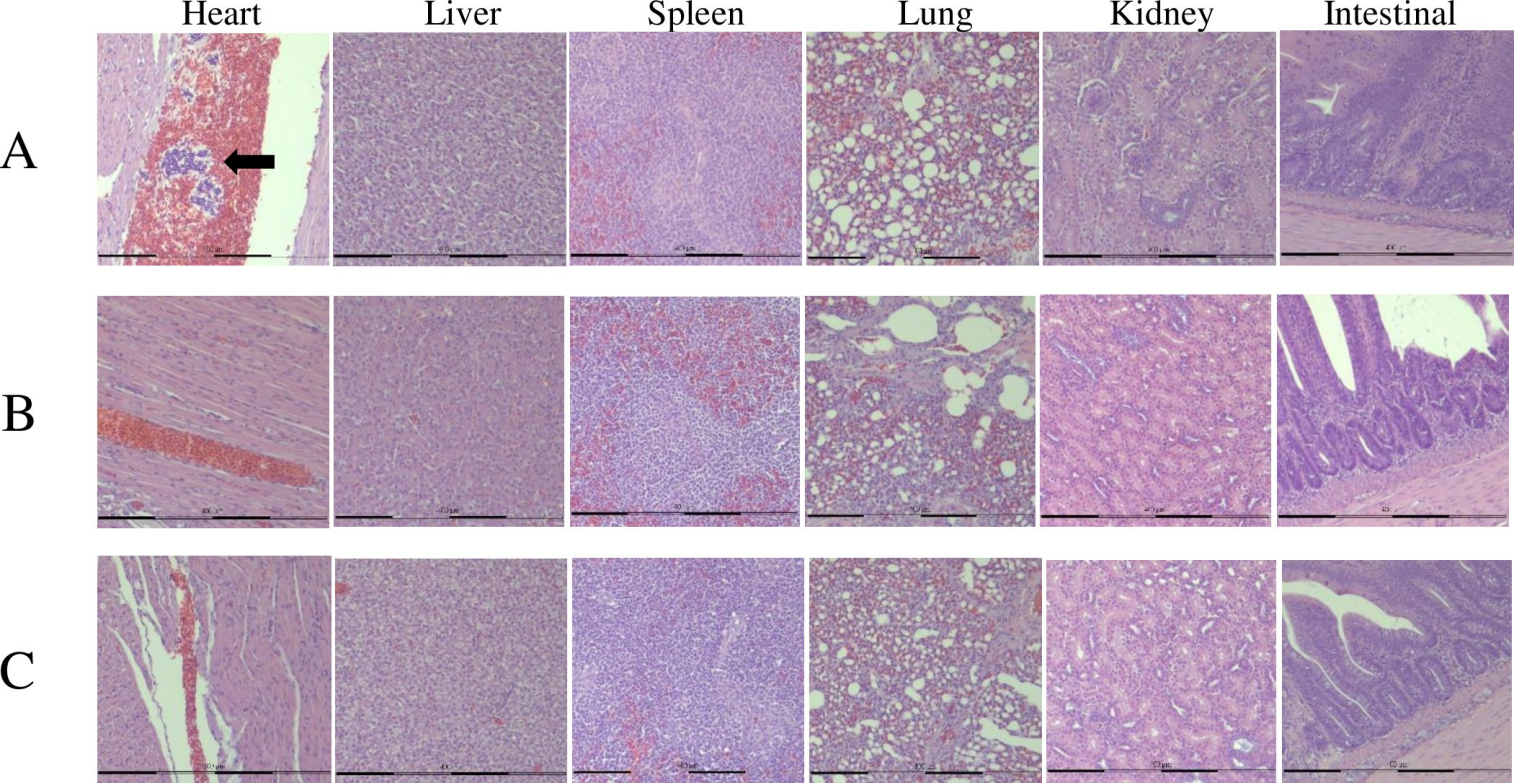
Histological analysis of tissue sections. Representative photomicrographs of tissue sections stained with H&E. A: Spiraline-A group (No. 50); B: 3% rTP4 group (No. 99); C: Basal diet group (No. 90).

**Fig 6:**
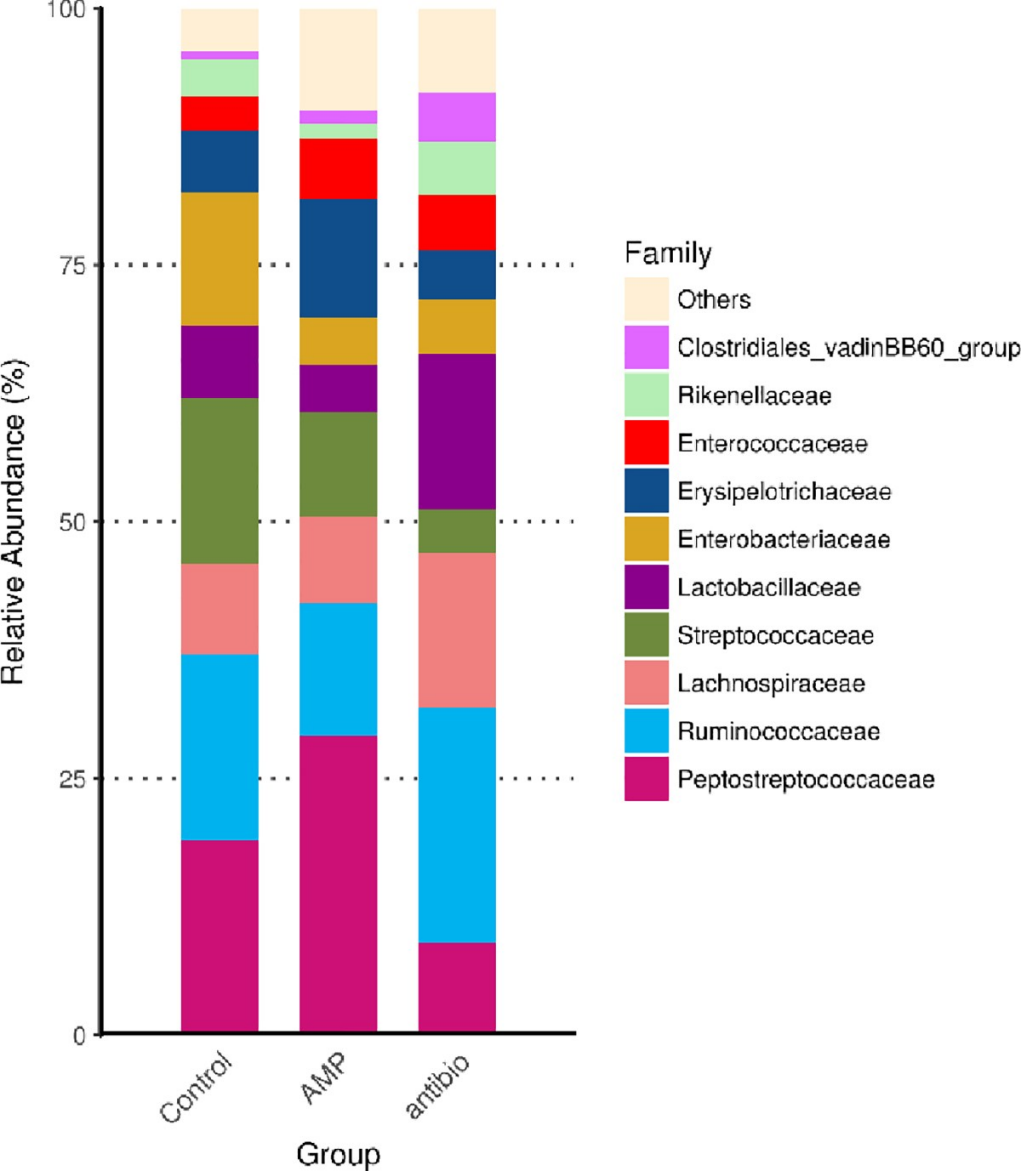
Effect of orally administered 3% rTP4, Spiraline-A and basal diet on the intestinal microflora in *G*. *g*. *domesticus*. Control: Basal diet group; AMP: 3% rTP4 group; antibio: Spiraline-A group.

After 36 days of the experimental period, the growth performance of *G*. *g*. *domesticus* in percent weight gain (WG) and feed efficiency (FE) in 3.0% rTP4-fed animals was significantly higher than that in the basal diet group animals and similar to that in the Spiraline-A group animals (Table 2). The protein efficiency ratios of *G*. *g*. *domesticus* in the 0.75% and 1.5% rTP4-fed animals were higher than those in the animals in other groups (Fig 4). The survival rate of animals fed 1.5% rTP4 was lower than that of the animals in other groups, which was caused by one chicken dying from disease and two chickens dying after pecking an infected carcass. Morphological measurements of the heart, liver, spleen, lung, kidney and intestine are given in Fig 5. Histological examination of tissues showed white blood cell aggregates in the coronary vein of the heart in the antibiotic group (Fig 5A, black arrow). This phenomenon was not found in the coronary veins of the 3% rTP4 group or basal diet group. Microscopic examination of tissue sections, including those of the heart, liver, spleen, lung, kidney and intestine, in the three groups was normal, and no other symptoms were observed. Microbial profiles of the duodenum in chickens with different treatments were determined using high-throughput sequencing of the bacterial 16S rRNA gene. The results showed that the duodenum was predominantly populated by *Peptostreptococcaceae* and *Erysipelotrichaceae*. Reduced total viable counts of *Ruminococcaceae* and *Lachnospiraceae* were also observed after feeding with rTP4 (Fig 6).

**Table 2:** Effect of weight gain ratio, feed efficiency (FE), protein efficiency ratio (PER) and survival rate of *G*. *g*. *domesticus* fed with diets containing rTP4 (0.75, 1.5, 3.0, 6.0 and 12%) spray-dried powder for 5 weeks^1^.

Group	Weight (Day 2)	Weight (Day 36)	Weight gain ^2^ (%)	FE ^3^	PER ^4^ (%)	Survival (%)
Spiraline-A (Commercially available)	47.86±0.90	1656.69±60.35	3373.51	0.53	2.44	82%
Basal diet	48.65±1.02	1542.51±84.30	3078.93	0.50	2.44	97%
0.75% rTP4	48.33±1.44	1504.33±101.79	3037.67	0.50	2.50	100%
1.5% rTP4	47.57±1.14	1639.93±98.71	3361.58	0.53	2.48	79%
3.0% rTP4	47.19±1.51	1639.25±75.15	3389.95	0.54	2.41	91%
6.0% rTP4	48.09± 1.24	1577.28±84.28	3192.45	0.51	2.27	85%
12.0% rTP4	48.71±1.20	1404.2±72.63	2789.93	0.49	2.26	100%

^1^ Values in the same column with different superscript are significantly different (p<0.05). Data are expressed as mean ± SD from group of chicken (n = 34).

^2^ Weight gain (%) = {Final body weight (g)–Initial body weight (g)}/Initial body weight (g) x 100.

^3^ Feed efficiency = {Final body weight (g)–Initial body weight (g)}/Feed intake (g).

^4^ Protein efficiency ratios = {Final body weight (g)–Initial body weight (g)}/Protein intake (g).

## 4. Discussion

Antibiotics are widely used to prevent pathogen infection and promote growth in poultry and livestock production [26]. However, many reports indicate that long-term use of low-dose antibiotics may lead to resistant microorganisms. For various reasons, the use of antibiotics has been banned or restricted for growth promotion in the poultry industry in many countries. Consequently, there is a high demand for antibiotic alternatives in the livestock industry. Compared with traditional antibiotics, AMPs have been shown to have multiple biological functions in addition to antimicrobial activities, including antibacterial, antiviral, antiprotozoan and antifungal activities [27]. In other respects, antimicrobial peptides protect fish against infections caused by the fish pathogen *Vibrio anguillarum* and provide a concept of transgenic fish with antimicrobial peptides. Antimicrobial peptides may decrease fish bacterial disease [28]. Antimicrobial peptides (AMPs) also stimulate the innate immune system via immunomodulatory functions and modulate the intestinal microflora in animals and humans [5]. In addition, AMPs had positive effects on intestinal morphology, nutrient digestibility, the gut microbiota, and growth performance in animals. These AMPs have a strong potential for application as a feed additive as alternatives to antibiotics in swine and poultry production. To study the role of antimicrobial peptides and mass production as feed supplements by use in the livestock industry, we performed this research. Yeast expression systems are ideally suited for large-scale production and cost-effective expression of functional AMP proteins. According to the codon usage preference of *P*. *pastoris*, the mature TP4 cDNA was modified, synthesized and cloned into the pPICZαA vector for *P*. *pastoris* X-33 expression.

There are many antimicrobial peptides that have been expressed in *P*. *pastoris* and possess natural antibacterial activities, such as ABP-dHC-cecropin A human neutrophil peptide 1 (HNP1) and histidalin [29–31]. In this study, we successfully produced recombinant TP4 on a 500-liter scale and freeze dried it to use as a feed additive for dietary supplementation in broilers. In the second cultivation, the viable count declined quickly from 8.3×10^10^ to 6.5×10^10^ CFU/mL, and the wet weight declined from 275 to 166 g/L during 49 to 58 hours of cultivation. This effect is presumably caused by methanol-induced antimicrobial peptide production to inhibit host cell growth [32]. According to previous research, the antimicrobial peptide HKPLP derived from *Hippocampus kuda* Bleeker showed good heat stability in thermal stability analysis [33]. After high-temperature treatment, rTP4 expressed by *P*. *pastoris* was found to be very stable *in vitro* and still retained full antimicrobial activity against *S*. *aureus* (BCRC 10780) and *E*. *coli* (BCRC 10675). The thermal stability of rTP4 may be related to its high arginine content (24%, 5 arginine in 25 total residues) [34]. In a previous report, increased arginine content enhanced peptide insertion into the membrane and significantly increased antimicrobial activity [35]. In addition, the antibacterial activity of AMPs can be enhanced by modifying the structural properties of natural peptides [36–38]. In a similar report, the crude antimicrobial extract of *Bacillus velezensis* strains isolated from stingless bee products showed bactericidal activity against methicillin-resistant *Staphylococcus aureus* and was stable at various temperatures (40–80°C) [39]. Furthermore, Plantaricin LD1 produced by *Lactobacillus plantarum* LD1 showed stability at high temperatures (100°C for 20 min and 121°C for 15 min under 15 psi) and inhibited bacteria such as *Staphylococcus aureus*, urogenic *Escherichia coli*, *Pseudomonas aeruginosa*, *Salmonella typhi*, *Shigella flexneri* and *Vibrio sp* [40]. Additionally, cecropinXJ expressed in *Escherichia coli* retained high stability against *Staphylococcus aureus* over a temperature range from 4 to 100°C [41].

These results show that compared to the basal diet, fodder supplementation with crude rTP4 can improve the growth of *G*. *g*. *domesticus*. Regarding the antibiotic group, the 1.5% rTP4 and 3.0% rTP4 groups were similar, indicating that the antimicrobial peptide rTP4 can be recommended as an alternative to antibiotics to promote growth performance in chickens. According to this study, the FCR of the basal diet group was around 2.0, the Spiraline-A (commercial) group was around 1.88, and the 3% rTP4-fed group was around 1.85. In our experience, typical FCRs for animals raised using commercial feeds and intensive production methods are as follows: beef cattle: 6.0–10.0, pigs: 2.7–5.0, farmed fish and shrimp: 1.0–2.4, and chickens: 1.7–2.0. The FCRs in this study range from 1.85 to 2.0, which is within the normal range for chickens. In previous research, similar to an antibiotic, the antimicrobial peptide cLF36 improved the immune cells, intestinal morphology, intestinal microbiome, junctional proteins, and growth performance in *E*. *coli*-challenged broilers [42]. Pigs fed AMP‐P5 at 60 mg kg^-1^ in dietary supplementation showed enhanced growth, inhibited coliform growth and increased nutrient digestion [43]. Tilapia piscidin, a marine antimicrobial peptide, has antimicrobial and immune regulatory activities that make it a potentially valuable feed supplement in aquaculture. Dietary supplementation with tilapia piscidin could enhance intestinal health, antioxidant activity, and the immune response. Furthermore, it confers protection against pathogen infections in Nile tilapia [44]. Morphological measurements of the heart, liver, spleen, lung, kidney and intestine showed white blood cell aggregates in the coronary vein of the heart in the broilers of the antibiotic group. The phenomenon of white blood cell aggregates is associated with infection or inflammation in the body. The Spiraline-A antibiotic group contained streptomycin sulfate. Streptomycin is administered by intramuscular injections in humans. Repeated injections of streptomycin can cause pain and inflammation [45]. Some reports have shown that the cecum mucosa of streptomycin-treated mice has mild inflammatory infiltration [46]. In the gut microbiota community richness analysis, the total viable counts of the *Ruminococcaceae* and *Lachnospiraceae* were reduced. *Peptostreptococcaceae* and *Erysipelotrichaceae* were enriched in the duodenum in the TP4 group compared with in the antibiotic and basal diet groups. A report showed an increase in *Lachnospiraceae* in WIRS mice, which is associated with human diseases, such as ulcerative colitis, celiac disease and Crohn’s disease [47].

### Conclusions

This study revealed that recombinant TP4 expressed by yeast has high antimicrobial activity and thermal stability. Therefore, rTP4 is available for feed application, as the antimicrobial peptide can withstand the high temperature used in pelleted feed procedure and still have strong antibacterial activity. These results showed that the antimicrobial peptide tilapia piscidin 4 can be used as a feed additive for the livestock industry.

## Supporting information

S1 Fig Plasmid map of the pPICZαA-TP4 vector. (PPT)

S2 Fig (TIF)

S1 Table Proximate analysis of basal feed with additive compositions at the early, middle and late stages. (DOC)

S2 Table Formulation of the basal diet at the early, middle and late stages. (DOC)
