# Supplementary figures and images for: Scale-up production of and dietary supplementation with the recombinant antimicrobial peptide tilapia piscidin 4 to improve growth performance in Gallus gallus domesticus
Source: PLoS One. 2021 Jun 24;16(6):e0253661. doi: 10.1371/journal.pone.0253661 (PMC8224963; doi:10.1371/journal.pone.0253661)

## Slide 1
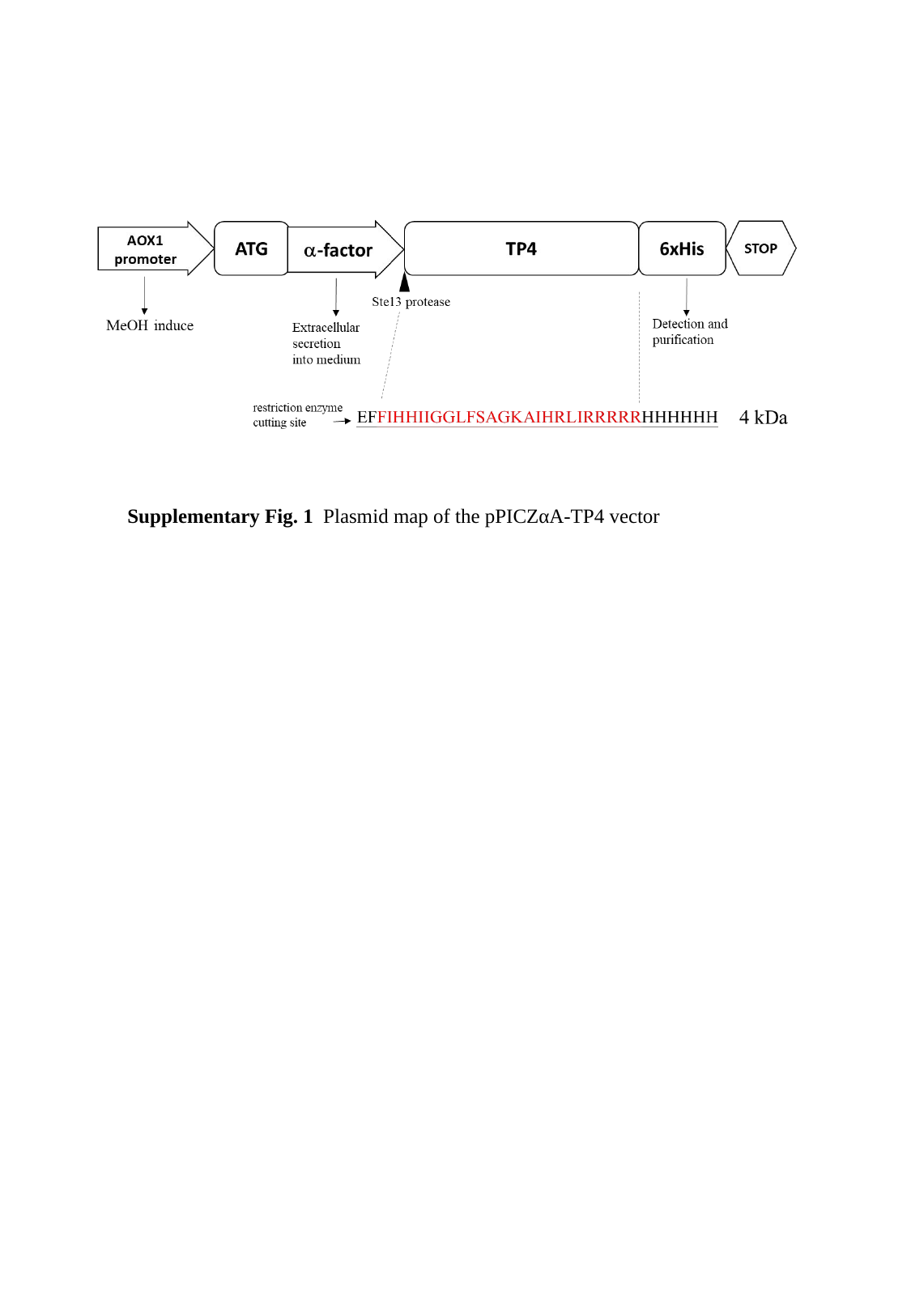

Supplementary Fig. 1 Plasmid map of the pPICZαA-TP4 vector

Supplement: S1 Fig — (PPT) [file pone.0253661.s001.ppt]

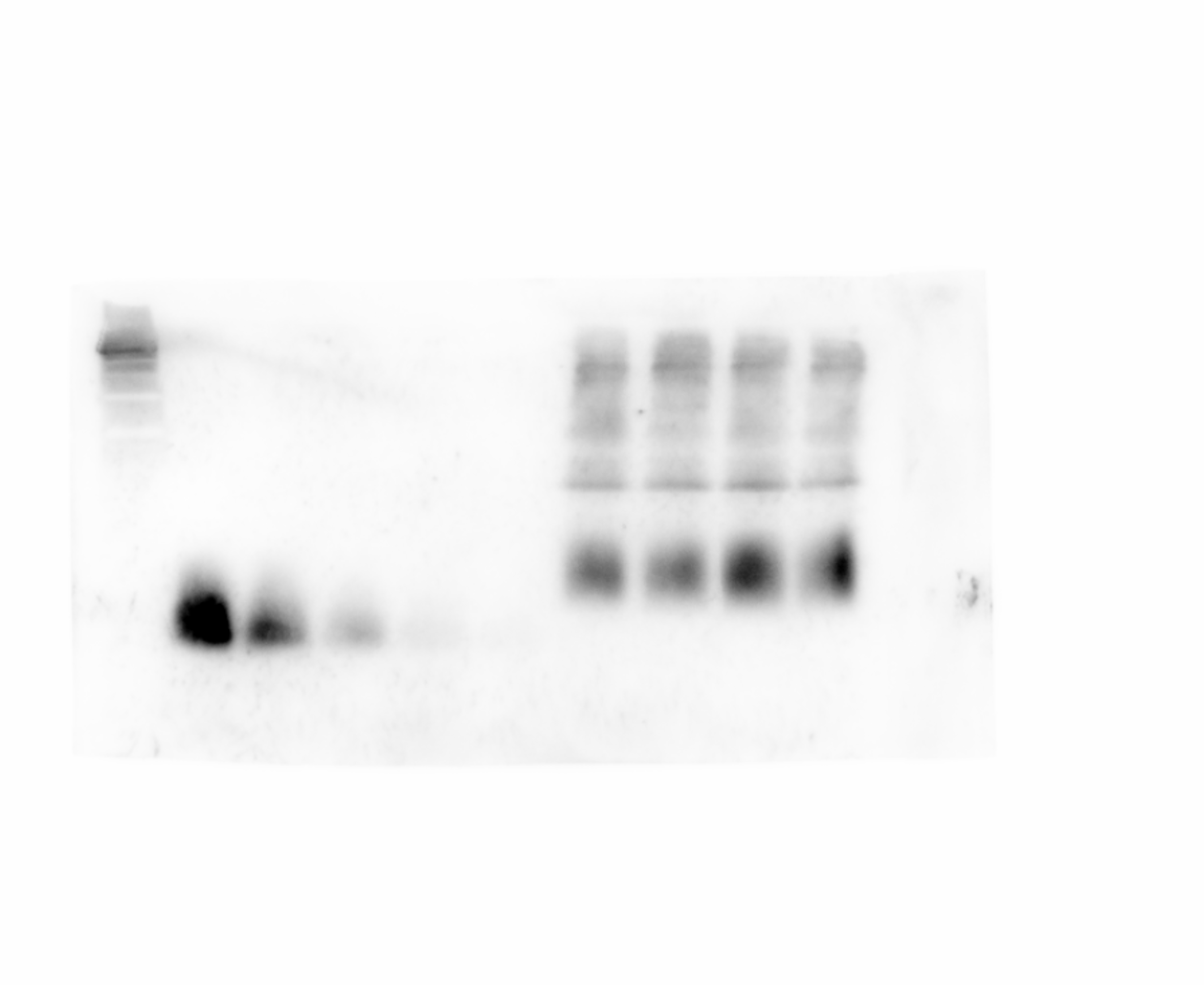

Supplement: S2 Fig — (TIF) [file pone.0253661.s002.tif]
