# Supplementary material for: Scale-up production of and dietary supplementation with the recombinant antimicrobial peptide tilapia piscidin 4 to improve growth performance in Gallus gallus domesticus
Source: PLoS One. 2021 Jun 24;16(6):e0253661. doi: 10.1371/journal.pone.0253661 (PMC8224963; doi:10.1371/journal.pone.0253661)
Supplement: S1 Table — (DOC) [file pone.0253661.s003.doc]

**Supplementary Table 1** Proximate analysis of basal feed with additive composition of early, middle and late stage.

**A**. Proximate analysis of basal feed with additive composition^1^ of early stage.

| Items (%) | Basal diet | 0.2% Antibiotic + extra vitamins and lysine^2^ | 0.75% rTP4 | 1.5% rTP4 | 3%  rTP4 | 6%  rTP4 | 12%  rTP4 |
| --- | --- | --- | --- | --- | --- | --- | --- |
| Crude protein | 19.91 | 20.81 | 19.09 | 22.43 | 21.03 | 20.01 | 18.95 |
| Crude lipid | 6.13 | 6.41 | 7.37 | 5.64 | 9.75 | 7.13 | 7.27 |
| Ash | 8.07 | 7.59 | 8.21 | 6.91 | 6.50 | 7.84 | 7.58 |

**B**. Proximate analysis of basal feed with additive composition of middle stage.

| Items (%) | Basal diet | 0.2% Antibiotic + extra vitamins and lysine^2^ | 0.75%  rTP4 | 1.5%  rTP4 | 3%  rTP4 | 6%  rTP4 | 12%  rTP4 |
| --- | --- | --- | --- | --- | --- | --- | --- |
| Crude protein | 20.39 | 17.24 | 22.41 | 19.53 | 20.11 | 20.47 | 21.17 |
| Crude lipid | 7.54 | 7.97 | 6.06 | 5.70 | 6.95 | 6.76 | 9.49 |
| Ash | 5.27 | 6.57 | 7.07 | 7.00 | 8.50 | 7.37 | 9.79 |

**C**. Proximate analysis of basal feed with additive composition of late stage.

| Items (%) | Basal diet | 0.2% Antibiotic + extra vitamins and lysine^2^ | 0.75% rTP4 | 1.5%  rTP4 | 3%  rTP4 | 6%  rTP4 | 12%  rTP4 |
| --- | --- | --- | --- | --- | --- | --- | --- |
| Crude protein | 17.83 | 14.82 | 17.90 | 20.52 | 18.23 | 16.84 | 18.41 |
| Crude lipid | 9.07 | 6.62 | 5.36 | 5.88 | 8.90 | 5.36 | 8.45 |
| Ash | 6.89 | 7.22 | 6.84 | 7.16 | 6.60 | 8.40 | 8.71 |

^1^ Fermentation supernatant spray-dried powder of TP4 was added to diets at a concentration of 0, 0.75, 1.5, 3.0, 6.0 or 12 g/100 g of diet.

^2^ Antibiotic (Spiraline-A) : each gram contains 30 mg of spiramycin adipate (pot.), 30 mg of streptomycin sulfate (pot.), 2,500 I.U. of vitamin A, 5 mg of vitamin B1, 10 mg of vitamin B2, 2 mg of vitamin B6, 5 µg of vitamin B12, 2 mg of vitamin E, 500 I.U. of vitamin D3, 1 mg of vitamin K4, 0.2 mg of folic acid, 5 mg of calcium pantothenic acid, 10 mg of nicotinic acid, 20 mg of lysine.
