# Supplementary material for: Scale-up production of and dietary supplementation with the recombinant antimicrobial peptide tilapia piscidin 4 to improve growth performance in Gallus gallus domesticus
Source: PLoS One. 2021 Jun 24;16(6):e0253661. doi: 10.1371/journal.pone.0253661 (PMC8224963; doi:10.1371/journal.pone.0253661)
Supplement: S2 Table — (DOC) [file pone.0253661.s004.doc]

**Supplementary Table 2** Formulation of the basal diet of early, middle and late stage

**A.** Formulation of the basal diet ^1^ of early stage.

| Ingredients (%) | Basal diet | 0.75% rTP4 | 1.5% rTP4 | 3% rTP4 | 6% rTP4 | 12% rTP4 | 0.2% Antibiotic + extra vitamins and lysine^2^ |
| --- | --- | --- | --- | --- | --- | --- | --- |
| Yellow corn | 54.3 | 54.3 | 54.3 | 54.3 | 54.3 | 54.3 | 54.3 |
| Soybean meal | 38 | 37.25 | 36.5 | 35 | 32 | 26 | 37.8 |
| Dicalcium phosphate | 1.4 | 1.4 | 1.4 | 1.4 | 1.4 | 1.4 | 1.4 |
| Calcium carbonate | 1 | 1 | 1 | 1 | 1 | 1 | 1 |
| Iodized salt | 0.3 | 0.3 | 0.3 | 0.3 | 0.3 | 0.3 | 0.3 |
| Soybean oil | 3 | 3 | 3 | 3 | 3 | 3 | 3 |
| Vitamin premix | 2 | 2 | 2 | 2 | 2 | 2 | 2 |
| Additive | 0 | 0.75 | 1.5 | 3 | 6 | 12 | 0.2 |

**B.** Formulation of the basal diet ^1^ of middle stage.

| Ingredients (%) | Basal diet | 0.75% rTP4 | 1.5% rTP4 | 3% rTP4 | 6% rTP4 | 12% rTP4 | 0.2% Antibiotic + extra vitamins and lysine^2^ |
| --- | --- | --- | --- | --- | --- | --- | --- |
| Yellow corn | 58.2 | 58.2 | 58.2 | 58.2 | 58.2 | 58.2 | 58.2 |
| Soybean meal | 34 | 33.25 | 32.5 | 31 | 28 | 22 | 33.8 |
| Dicalcium phosphate | 1.4 | 1.4 | 1.4 | 1.4 | 1.4 | 1.4 | 1.4 |
| Calcium carbonate | 1.1 | 1.1 | 1.1 | 1.1 | 1.1 | 1.1 | 1.1 |
| Iodized salt | 0.3 | 0.3 | 0.3 | 0.3 | 0.3 | 0.3 | 0.3 |
| Soybean oil | 3 | 3 | 3 | 3 | 3 | 3 | 3 |
| Vitamin premix | 2 | 2 | 2 | 2 | 2 | 2 | 2 |
| Additive | 0 | 0.75 | 1.5 | 3 | 6 | 12 | 0.2 |

**C.** Formulation of the basal diet ^1^ of late stage.

| Ingredients (%) | Basal diet | 0.75% rTP4 | 1.5% rTP4 | 3% rTP4 | 6% rTP4 | 12% rTP4 | 0.2% Antibiotic + extra vitamins and lysine^2^ |
| --- | --- | --- | --- | --- | --- | --- | --- |
| Yellow corn | 60.2 | 60.2 | 60.2 | 60.2 | 60.2 | 60.2 | 60.2 |
| Soybean meal | 29 | 28.25 | 27.5 | 26 | 23 | 17 | 28.8 |
| Dicalcium phosphate | 1.3 | 1.3 | 1.3 | 1.3 | 1.3 | 1.3 | 1.3 |
| Wheat bran | 3 | 3 | 3 | 3 | 3 | 3 | 3 |
| Calcium carbonate | 1.2 | 1.2 | 1.2 | 1.2 | 1.2 | 1.2 | 1.2 |
| Iodized salt | 0.3 | 0.3 | 0.3 | 0.3 | 0.3 | 0.3 | 0.3 |
| Soybean oil | 3 | 3 | 3 | 3 | 3 | 3 | 3 |
| Vitamin premix | 2 | 2 | 2 | 2 | 2 | 2 | 2 |
| Additive | 0 | 0.75 | 1.5 | 3 | 6 | 12 | 0.2 |

^1^ Fermentation supernatant spray-dried powder of TP4 was added to diets at a concentration of 0, 0.75, 1.5, 3.0, 6.0 or 12 g/100 g of diet.

^2^ Antibiotic: each gram contains 30 mg of spiramycin adipate (pot.), 30 mg of streptomycin sulfate (pot.), 2,500 I.U. of vitamin A, 5 mg of vitamin B1, 10 mg of vitamin B2, 2 mg of vitamin B6, 5 µg of vitamin B12, 2 mg of vitamin E, 500 I.U. of vitamin D3, 1 mg of vitamin K4, 0.2 mg of folic acid, 5 mg of calcium pantothenic acid, 10 mg of nicotinic acid, 20 mg of lysine.
